# The unique monoclonal antibodies and immunochemical assay for comprehensive determination of the cell-bound and soluble HER2 in different biological samples

**DOI:** 10.1038/s41598-024-54590-z

**Published:** 2024-02-17

**Authors:** Aleksandra Antos, Agnieszka Topolska-Woś, Marcin Woś, Agata Mitura, Paulina Sarzyńska, Tomasz Lipiński, Andrzej Kurylcio, Piotr Ziółkowski, Marta Świtalska, Joanna Tkaczuk-Włach, Andrzej Gamian, Wojciech P. Polkowski, Magdalena Staniszewska

**Affiliations:** 1 SDS Optic, EcoTech Complex, Block A, Głęboka 39, 20-612 Lublin, Poland; 2 Lukasiewicz Research Network – PORT Polish Center for Technology Development, Stabłowicka 147, 54-066 Wrocław, Poland; 3 Department of Surgical Oncology, Medical University of Lublin, Radziwiłłowska 13, 20-080 Lublin, Poland; 4 Department of Pathomorphology, Wrocław Medical University, Marcinkowskiego 1, 50-368 Wrocław, Poland; 5 Department of Experimental Oncology, Hirszfeld Institute of Immunology and Experimental Therapy, Polish Academy of Sciences, Weigla 12, 53-114 Wrocław, Poland; 6 Chair of Obstetrics and Gynecology, Faculty of Health Sciences, Medical University of Lublin, Staszica 4/6, 20-081 Lublin, Poland; 7 Laboratory of Medical Microbiology, Hirszfeld Institute of Immunology and Experimental Therapy, Polish Academy of Sciences, Weigla 12, 53-114 Wrocław, Poland; 8 Faculty of Medicine, The John Paul II Catholic University of Lublin, Konstantynów 1J, 20-708 Lublin, Poland

**Keywords:** Immunochemistry, Biomarkers, Tumour biomarkers

## Abstract

The expression of the HER2 (human epidermal growth factor receptor 2) protein in cancer cells is a well-established cancer marker used for diagnostic and therapeutic purposes in modern treatment protocols, especially in breast cancer. The gold-standard immunohistochemical diagnostic methods with the specific anti-HER2 antibodies are utilized in the clinic to measure expression level of the membrane-bound receptor. However, a soluble extracellular domain (ECD) of HER2 is released to the extracellular matrix, thus the blood assays for HER2 measurements present an attractive way for HER2 level determination. There is a need for accurate and validated assays that can be used to correlate the concentration of the circulating HER2 protein with disease clinical manifestations. Here we describe two monoclonal antibodies binding HER2 with a unique sequence of the complementarity-determining regions that recognize HER2 ECD. Development and validation of the sandwich enzyme-linked immunosorbent assay (ELISA) for quantification of the soluble HER2 in a variety of biological samples is also presented. The assay provides HER2 quantitation within a concentrations range from 1.56 to 100 ng/ml with sensitivity at the level of 0.5 ng/ml that meets the expectations for measurements of HER2 in the blood and tumor tissue samples. The method presents satisfactory intra- and inter-assay precision and accuracy for immunochemical quantification of biomarkers in biological samples. The utility of the generated monoclonal anti-HER2 antibodies has been confirmed for use in the precise measurement of HER2 (both cell-bound and soluble) in several types of biological material, including serum, solid tumor tissue, and cell culture medium. Additionally, the developed immunochemical tools have a potential for HER2 detection, not only in a wide range of sample types but also independently of the sample storage/pre-processing, allowing for comprehensive HER2 analysis in tissue (IHC), cultured cells (immunofluorescence) and blood (ELISA).

## Introduction

HER2 belongs to a large family of EGFR receptors and in the cell is expressed as a proto-oncogene. The protein receptor with a molecular mass of 185 kDa carries tyrosine kinase activity and is located in the cell membrane. The full-length receptor is composed of three domains—the extracellular domain (ECD), the hydrophobic transmembrane domain, and the intracellular domain with tyrosine kinase activity. In the pathological states, the HER2 receptor is overexpressed and activated, resulting in the aggressive form of the disease. Its elevated expression is detected in up to 30% of breast cancers and typically contributes to a poor prognosis^1^. Several studies have also investigated the clinical significance of the HER2 overexpression in other cancers including stomach, ovary, endometrium, bladder, lung, colon, and head and neck. HER2 receptor can be shed as the HER2 ECD and released into the extracellular matrix^2^ in the form of a soluble receptor (sHER2). While the level of this biomarker in the direct surrounding of the tumor increases, a significantly elevated amount of sHER2 can be detected also in circulation presenting a valuable predictive and prognostic marker^3,4^.

Several HER2-targeting agents are available with confirmed benefits in patients with HER2-positive cancers. As treatment with trastuzumab, pertuzumab, lapatinib, or trastuzumab emtansine can be applied upon confirmation of the HER2 positive status, thus an accurate detection is critical. Currently, two methods have been approved and widely used for HER2 routine testing. HER2 overexpression is tested using immunohistochemistry (IHC) and *HER2* gene amplification by fluorescence in situ hybridization (FISH). Additionally, the Oncotype DX assay is used for HER2 mRNA expression. Since both approved methods rely on tumor tissue biopsy, the performance and result interpretation are potential source of bias, making determination of patient eligibility for appropriate treatment and their potential responsiveness to therapies uncertain. The American Society of Clinical Oncology (ASCO) and the College of American Pathologists (CAP) in 2007 developed guidelines updated in 2018 to set standards and reduce the variability between the testing units. Nevertheless, HER2 concordance remains a subject for improvement^5^.

Enzyme-linked immunosorbent assay (ELISA) is a broadly used analytical technique for the detection of the target protein present in a given sample. It relies on the specific antibodies binding the desired molecule. There are several types of this method, including direct and indirect, sandwich, competitive, inhibitory, etc. The assay in contrast to IHC or FISH, which require solid tissue biopsy, this assay enables the accurate detection of a soluble protein in the easily accessible blood- and liquid-based biological material (so called liquid biopsy). The most commercially available ELISAs for quantification of a soluble HER2 are dedicated to research only, including the assay provided by R&D Systems, Inc. (USA, Minneapolis). There is a limited number of FDA-approved assays, like the one available in a microplate format produced at the IVD grade by Siemens Healthineers^6^ (Erlangen, Germany). It has been suggested in the available reports that circulating HER2 ECD blood levels could be used as a surrogate marker, especially in the context of the treatment response in metastatic breast cancer (MBC)^7^ or as a complementary method to IHC to identify patients eligible to anti-HER2 treatment^8^. While there is a controversy over the utility of the HER2 ECD diagnostics^2^, the approved cut-off value for increased sHER2 concentration is 15 ng/ml^9^ according to the Food and Drug Administration and various manufacturer’s (e.g. Siemens Healthineers, Bayer) recommendations. Moderate sHER2 increase (up to 50 ng/ml) has also been described in other conditions, like such as liver disease, preeclampsia, and chronic heart failure^10–12^. Despite ELISA for HER2 ECD detection being an attractive approach, its context of use may not be universally accepted, thus more research and validated assays with uniformly accepted thresholds are needed to establish new diagnostic standards of care.

More work is being done on epitope mapping to better characterize the utilized anti-HER2 antibodies and to validate the applicability of the assays^13^. In this report, we present the unique sequence of newly developed and characterized monoclonal antibodies (mAb) binding the ECD domain of the HER2 receptor, independently of its protein native or denatured state. A detailed protocol and validation of the in-house sandwich ELISA designed to quantify HER2 expression is described. The performance of the assay has been tested on biological samples of different origins, including in vitro cultured cells, serum, and tissues. The immunochemical tools will contribute to the research, diagnostics, and development of novel anti-HER2 treatments in the future.

## Results

### Anti-HER2 antibody development and characterization

**Figure 1:**
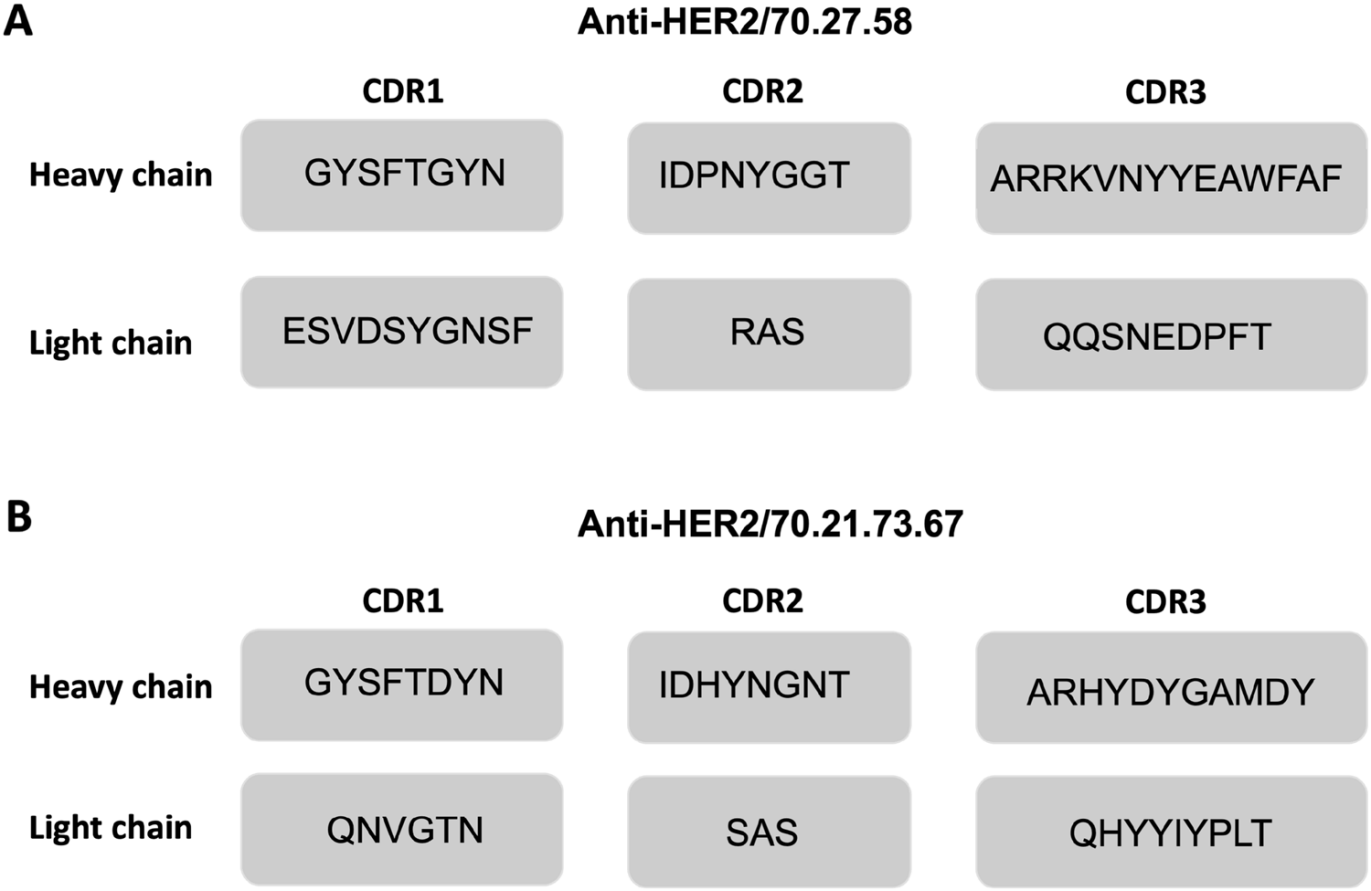
Unique fragments of the CDRs within the heavy and light chains of the new mouse monoclonal anti-HER2 antibody. The amino acid sequence corresponds to the characteristic nucleotide sequence of **(A**) anti-human HER2/70.27.58 mAb and **(B**) anti-human HER2/70.21.73.67 mAb.

New anti-HER2 antibodies were generated using the hybridoma cells technology utilizing the previously described method^14^. The selection of cells producing the desired antibody was performed using the N-terminal portion of the extracellular domain of a native HER2 receptor (residues Thr23—Ala510) and finally, two of the best-performing clones, named 70.27.58 and 70.21.73.67, were chosen for further research. Sequencing of the hybridomas’ DNA confirmed the presence of the domains corresponding to a heavy chain of immunoglobulin $\upgamma$ and a light chain of the $\upkappa$ protein (HER2/70.27.58; GenBank: OR400146, OR400147 and HER2/70.21.73.67; GenBank: OR400148 and OR400149). Sequence analysis showed the unique nature of the selected anti-HER2 antibodies revealed several differences in the light and heavy chain complementarity-determining regions (CDRs) in comparison to the known anti-HER2 antibodies (Fig. 1).

**Figure 2:**
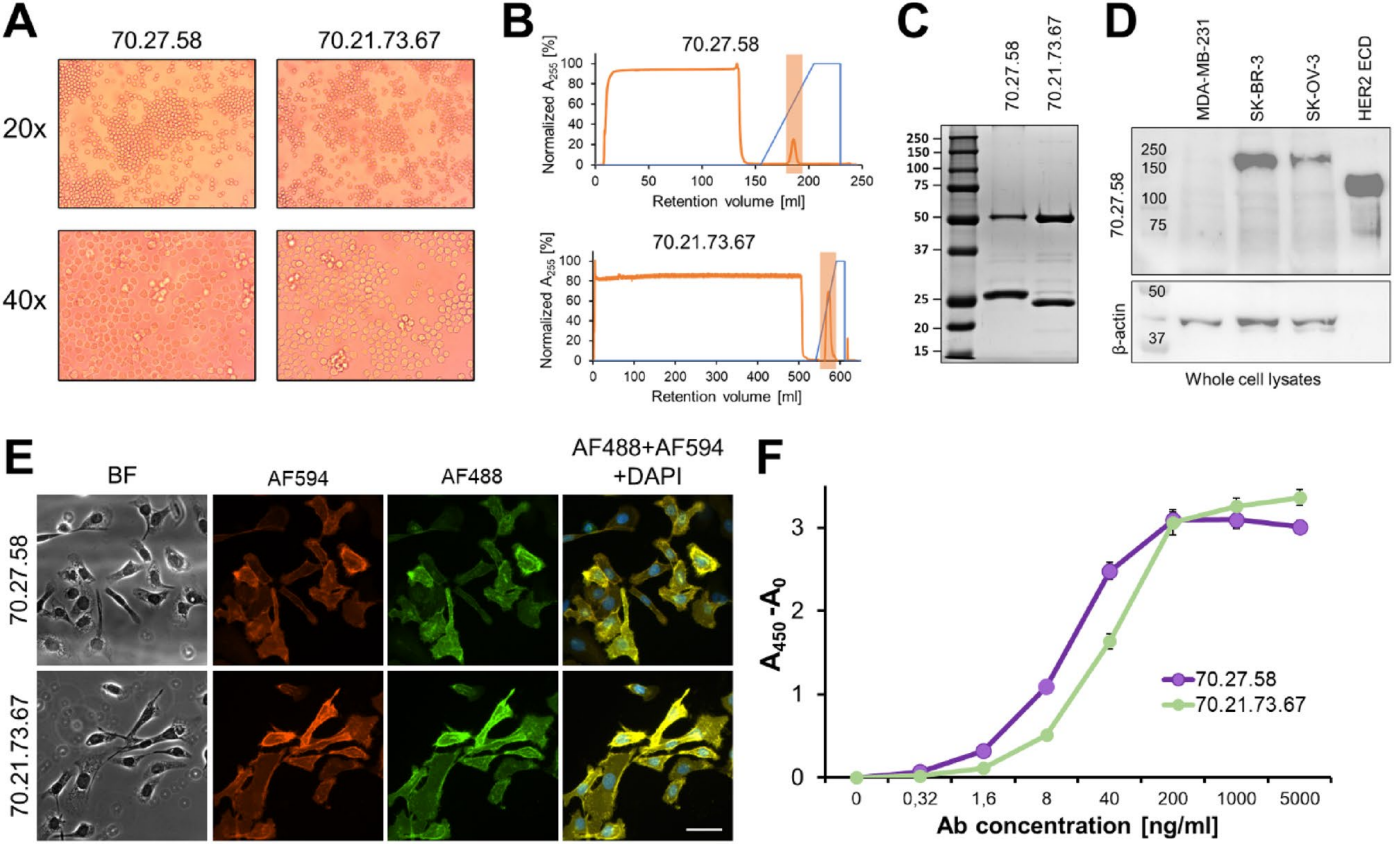
Anti-HER2 monoclonal antibodies production and characterization. (**A**) Morphology of the anti-human HER2/70.27.58 and anti-human HER2/70.21.73.67 hybridoma cells photographed at 20 × and 40 × magnification. (**B**) FPLC chromatograms were recorded during the purification of the anti-human HER2/70.27.58 and anti-human HER2/70.21.73.67 antibodies using affinity chromatography on the Protein A resin. (**C**) SDS-PAGE analysis of the purified anti-human HER2/70.27.58 and anti-human HER2/70.21.73.67 antibodies loaded at the amount of 1 µg/well on the 12% polyacrylamide gel under reducing conditions. (**D**) WB analysis of HER2 in whole cell lysates of the HER2 low expressing (MDA-MB-231) and HER2 high expressing (SK-BR-3, SK-OV-3) cells, probed with the home-made anti-human HER2/70.27.58 monoclonal antibody and detected with the secondary anti-mouse IgG-HRP (upper panel). The recombinant HER2 ECD protein was used as a reference. The loading control was performed with membrane probed with antibody binding $\upbeta$-actin (lower panel). (**E**) The formaldehyde-fixed SK-OV-3 cells were photographed in the bright field (BF) at the 40 × magnification. Immunofluorescence analysis was performed on cells stained with the commercial anti-HER2 ECD antibody followed by anti-mouse IgG-AlexaFluor594 (AF594) (red channel) and co-stained with the anti-HER2/70.27.58 or anti-HER2/70.21.73.67 antibodies detected with the AlexaFluor488-labeled (AF488) secondary antibody (green channel). Nuclei were stained with DAPI (blue channel) (**F**) Quantitative ELISA with the anti-human HER2/70.27.58 and anti-human HER2/70.21.73.67 antibodies loaded in a range of 0–5 µg/ml on the plate coated with the recombinant chimera of the HER2 ECD-Fc protein. The signal generated from secondary antibody anti-mouse IgG-HRP was quantified by measuring absorbance at 450 nm and expressed after background subtraction (A_450_-A_0_).

The monoclonal antibodies secreted by the generated hybridoma cells (Fig. 2A) were further purified from the collected post-culture medium by affinity purification using Fast Protein Liquid Chromatography (FPLC) system on the Protein A resin (Fig. 2B). The production yielded on average 3.98 mg ± 2.99 and 2.92 mg ± 1.6 of protein for anti-human HER2/clone 70.27.58 and anti-human HER2/clone 70.21.73.67 pure antibody, respectively from 1 L of the harvested medium. The protein recovery, assessed by densitometric analysis of an SDS-PAGE band (data not shown), reached up to 99%. The purified antibody showed a characteristic protein bands pattern, corresponding to the domains of light and heavy chains under reducing conditions (Fig. 2C), however clones 70.27.58 and 70.21.73.67 differed in light chain mass, migrating on polyacrylamide gel as bands of > 25 kDa and < 25 kDa, respectively. The antibody immunoreactivity was tested in Western Blotting (WB) with cell lysate of control and overexpressed level of HER2 receptor, namely MDA-MB-231 and SK-OV-3, SK-BR-3 cell lines, respectively (Fig. 2D). The generated anti-HER2 antibodies recognize the epitope on both the denatured full-length HER2 protein from the whole cell lysates (SK-BR-3, SK-OV-3) of human cancer cells or recombinant chimeric protein HER2 ECD-Fc (Fig. 2D) and the antigen in native conformation as analyzed on the SK-OV-3 cells (HER2^+^) by immunofluorescence (IF; Fig. 2E) and ELISA (Fig. 2F). Specificity of antibodies toward ECD of a human HER2 protein was also confirmed by co-staining with commercially available antibody counterparts generated in rabbits (Fig. 2E, panel AF594 and Fig. S1, panel A). The signal from the different antibodies, i.e. home-made and commercial ones co-localized resulting in a yellow signal (panels AF488 + AF594 + DAPI) suggesting binding the same antigen present within the HER2-overexpressing cells (SK-OV-3). In contrast, on MDA-MB-231 cells that show low HER2 expression much lower signal was observed when anti-HER2 antibodies were used for staining, regardless of antibody origin (Fig. S1, panel B). The minimal background signal resulting from the secondary antibody in the red or green channel (Fig. S1, panel B) also indicates good specificity of the homemade mAb toward HER2.

Finally, the affinity of the anti-human HER2/clone 70.27.58 or anti-human HER2/clone 70.21.73.67 antibodies for the antigen was established by direct ELISA on the plate coated with the recombinant HER2 ECD protein containing C-terminal poly-Histidine tag. The results showed strong but different activity of the tested antibodies (Fig. 2F) with ED50 corresponding to 0.0922 nM and 0.2347 nM for HER2/clone 70.27.58 and HER2/clone 70.21.73.67, respectively.

### Assay development and optimization

#### Assay design and assembly

The sandwich ELISA was designed for direct measurement of the human HER2 protein level with a unique combination of the different epitope-specific primary antibodies, i.e. HER2/clone 70.27.58 and HER2/clone 70.21.73.67. Based on ED50 values clone 70.27.58 was chosen for plate coating serving as the capturing antibody, while clone 70.21.73.67 was used as a detecting antibody in the ELISA sandwich. A schematic diagram of the assay is presented in Fig. S2.

As a result of optimization, due to the widest detection range and the best signal/noise ratio, the 0.1 µg/well of capturing antibody specific to HER2 was chosen for plate coating. Additionally, two commercial anti-human HER2 monoclonal antibodies were tested, however, none of them formed a functional complimentary pair required for a sandwich assay (data not shown).

Since a blocking agent fills the free space of the plate upon coating, the blocking step in the assay remains a critical factor in reducing a non-specific binding of sample components, thereby improving a signal/noise ratio. Based on the results for the mocked samples of recombinant antigen, 5% non-fat dry milk (NFDM) in PBS with 0.05% Tween-20 (PBST) solution was chosen for subsequent experiments due to a lower background level and better reproducibility of the assay. Next, the labeling (biotinylation) of the second (detection) antibody, namely anti-human HER2/70.21.73.67 mAb was optimized. Regardless of the type of biotin-labeling kit, no significant influence on the reproducibility of the results on mocked samples, sensitivity, or signal/noise ratio was observed. After a comparison of the two enzyme-conjugated detection reagents, the best results were obtained for avidin-HRP conjugate at 1:40 000 dilution in PBS and this agent was selected for use in the subsequent analysis. Finally, the effect of temperature was tested, and the results observed at 37 °C showed the best cofactor variability CV% (< 10%) and linearity R^2^ (0.998).

**Figure 3:**
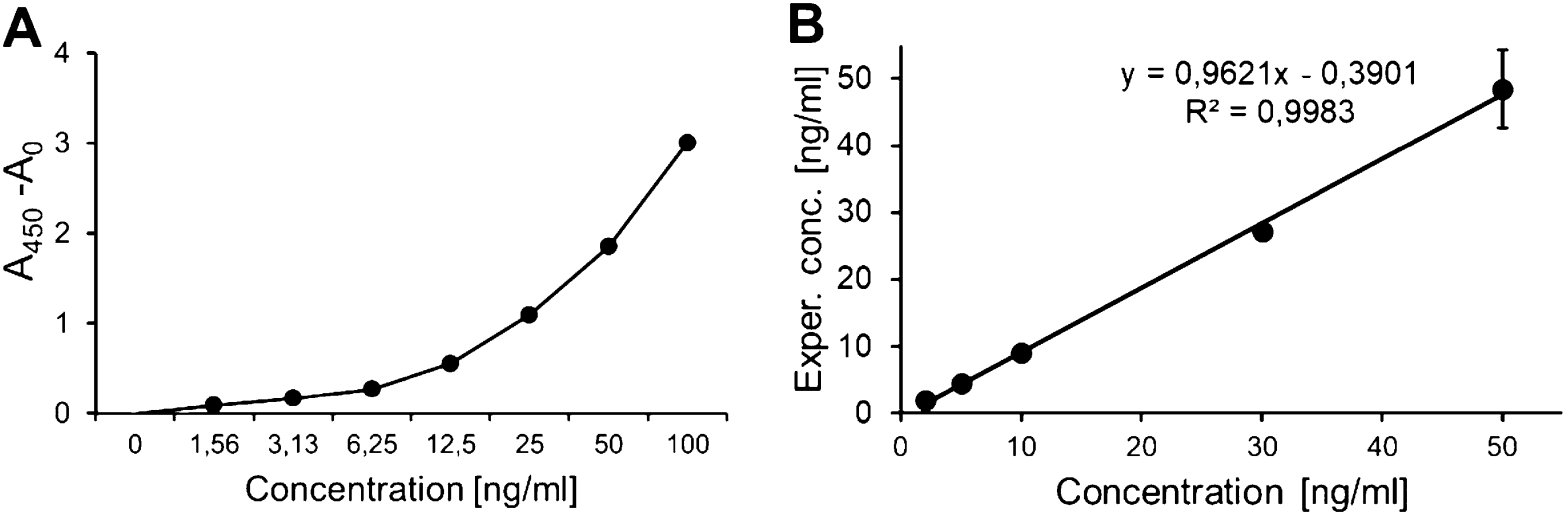
Parameters of the sandwich ELISA for HER2 detection. (**A**) HER2 binding kinetics in the standard curve concentration range of 0.156–10 000 ng/well (1.56–100 ng/ml). Results are expressed as absorbance at 450 nm after background subtraction (A_450_-A_0_). (**B**) Assay accuracy was tested by comparison of HER2 level measured by ELISA in the samples of the known antigen concentration (mock samples). Data were collected for 2, 5, 10, 30, and 50 ng/ml of HER2 (given concentration; *x-axis*), covering both physiological and increased concentrations. Experimentally measured concentration [ng/ml] is shown on the *y-axis*. Error bars indicate SD.

Overall, the in-house ELISA at the conditions chosen based on a multi-step optimization, represents a good performance within the standard curve range up to 100 ng/ml, ensuring the correct assembly of the complementary pair of antibodies binding the HER2 antigen in a sandwich assay (Fig. 3A).

#### Assay validation: sensitivity, specificity, precision

Assay linearity was determined on mocked samples at 3 levels of concentrations, i.e. low, medium, and relatively high 50 ng/ml concentrations of the HER2 in PBS. Linearity of the dose–response signal showed R^2^ correlation coefficient within a range of 0.98–1.00 (Fig. 3B). The coefficient of intra- and inter-assay variation among at least 12 assays on different days presented satisfactory precision of the assay with values lower than 10% and 25%, respectively.

The lower limit of detection (LLOD) was determined to be 0.5 ng/ml in PBS and human serum.

Testing for the lower limit of quantification and upper limit of quantification (LLOQ and ULOQ) conducted for two matrices, namely PBS and commercial pooled human serum demonstrated satisfactory accuracy and precision in good agreement with the range of a standard curve working range. Using the required dilution of human serum (2 times), the obtained LLOQ is 1.2 ng/ml and 112.7 ng/ml for ULOQ.

#### Spike and recovery (SAR) assessment

The Spike and Recovery (SAR) evaluation was performed for ELISA accuracy assessment and to determine the matrix effect. Known concentrations of recombinant HER2 ECD were dissolved in 7 different types of matrices, including PBS, pooled human serum (HS) of the healthy volunteers, Fetal Bovine Serum (FBS), as well as medium and whole cell lysate of MDA-MB-231 and SK-OV-3 cells (Table 1). Each matrix was tested individually, without a spike for reference. During the first testing, excessive concentration was determined for spiked samples, in human serum as a matrix. A two-fold dilution of human serum was employed to avoid matrix interferences caused by the cross-reactivity of an unknown factor with the capture antibody. The obtained result was satisfying. Among the tested matrices reported in Table 1, most results fit within an accepted recovery range of 80–120%^15^. Some results, mainly using SK-OV-3 whole cell lysate and FBS as a matrix land above the accepted range, suggesting an additional factor that interferes with the assay.

**Table 1:** The Spike and Recovery assay for different biological matrices.

Matrix	Spike level (ng/well)	Experimental average (ng/well)	Recovery (%)
PBS	0	0	–
	2	2	100.0
	5	4.56	91.1
	10	9.18	91.8
	30	27.14	90.5
	50	48.49	97.0
HS (1:2 dilution)	0	0.92	–
	2	1.33	66.3
	5	5.24	104.9
	10	12.95	129.5
	30	33.97	113.2
	50	59.88	119.8
FBS	0	0	–
	2	0	0
	5	5.12	102.4
	10	14.82	148.2
	30	49.31	164.4
	50	74.13	148.3
MDA-MB-231 whole-cell lysate (1:2000 dilution)	0	0.09	–
	2	3.06	153
	5	4.42	88.3
	10	11.22	112.2
	30	29.08	96.9
	50	44.17	88.3
MDA-MB-231 medium	0	0.25	–
	2	2.99	149.3
	5	5.71	114.1
	10	9.84	98.4
	30	29.9	99.7
	50	49.13	98.3
SK-OV-3 whole cell lysate (1:2000 dilution)	0	0.03	–
	2	2.42	121
	5	6.83	136.5
	10	12.76	127.6
	30	37.4	124.7
	50	59.04	118.1
SK-OV-3 medium	0	0.14	–
	2	1.49	74.5
	5	5.9	117.9
	10	11.83	118.3
	30	36.47	121.6
	50	58.11	116.2

The Spike level represents the recombinant HER2 concentration in different matrices. Recovery is calculated based on the experimental average and HER2 level in a standard curve diluent (PBS). The dilution of a particular matrix is provided in brackets.

*Reference recovery range: 80–120%^15^.

#### Assay stability

Addressing a need for long-term storage of an enzyme-based reagent system, leaving a great potential for use in point-of-care devices, we adapted the classical sandwich ELISA protocol to a storage of the pre-coated plate for the duration of up to 4 months. Following the developed protocol for a plate coated with anti-human HER2/70.27.58 mAb and the blocking step, the plate was incubated with trehalose in the blocking solution and dried out. Storage at 4 °C in humidity-controlled conditions for 4 months did not affect assay activity. Consistent ELISA performance was tested using mock samples containing recombinant HER2 ECD protein, within 6 months and 30-day intervals, starting from the first day since plate coating. Results confirmed no loss of the antibody-bound peroxidase activity within 120 days (4 months) from the plate coating. Analyses performed upon 150 and 180 days (5 and 6 months) of plate storage resulted in the intra-assay CV% of sample replicates exceeding the established criterion of ≤ 10%.

### Detection of HER2 in different biological samples from humans and mice

#### *Determination of HER2 released from human cells cultured *in vitro

The innovative ELISA was utilized for determination of HER2 level in the human cancer cells and the culture medium of lines with a confirmed increased expression of HER2 (SK-OV-3 and SK-BR-3) and MDA-MB-231 cells with low HER2 expression rather than completely lacking the receptor^4^. ELISA Quantification of HER2 expressed on the cells and secreted into the culturing medium was performed using the human cancer cells grown at the standard conditions. Both culture medium and cell mass (RIPA lysates) were collected at three time points: 24, 48, and 72 h. As expected, an increased level of HER2 (maximal measured in the 72 h-cultures) was detected both in the medium (25.68–145.75 ng/ml) and the HER2 overexpressing cell (SK-OV-3 and SK-BR-3) lysates (21.1–22.9 $\upmu$g/ml), with MDA-MB-231 levels being significantly lower (12.56 ng/ml and 14 $\upmu$g/ml, respectively for medium and cell lysates). Importantly, the level of the full-length receptor from RIPA lysates was found to be 1 000-fold higher than in the medium (ng/ml for medium vs $\upmu$g/ml in cell lysates) (Table 2), which can be associated with a significant dilution of the sHER2 in the culture medium.

**Table 2:** HER2 levels in the cell culture medium and whole cell lysates of MDA-MB-231, SK-OV-3, and SK-BR-3 cells measured by in-house ELISA.

Cell line	Sample type	Growth time	HER2 [ng/ml]
MDA-MB-231	Medium	24 h 48 h 72 h	1.28 (9.3%) 6.20 (7.5%) 12.56 (5.2%)
	Whole-cell lysate	24 h 48 h 72 h	3 700.59 (3%) 13 783.75 (6.8%) 14 005.23 (7.9%)
SK-OV-3	Medium	24 h 48 h 72 h	9.79 (4.1%) 18.97 (5.1%) 25.68 (2%)
	Whole-cell lysate	24 h 48 h 72 h	9 717.43 (7.6%) 13 827.54 (5.4%) 21 130.25 (5.9%)
SK-BR-3	Medium	24 h 48 h 72 h	63.22 (3.8%) 102.38 (2.2%) 145.75 (2.4%)
	Whole-cell lysate	24 h 48 h 72 h	17 181.01 (5.5%) 31 770.42 (6.8%) 22 896.74 (3.4%)

Samples of cell culture medium and cells for the whole cell lysates in RIPA buffer were harvested at 24, 48, and 72 h. HER2 concentrations (ng/ml) represent the average value of 3 replicates along with the coefficient of variation shown in brackets (%CV).

#### HER2 determination in serum and tumor tissues from xenografted mice

**Figure 4:**
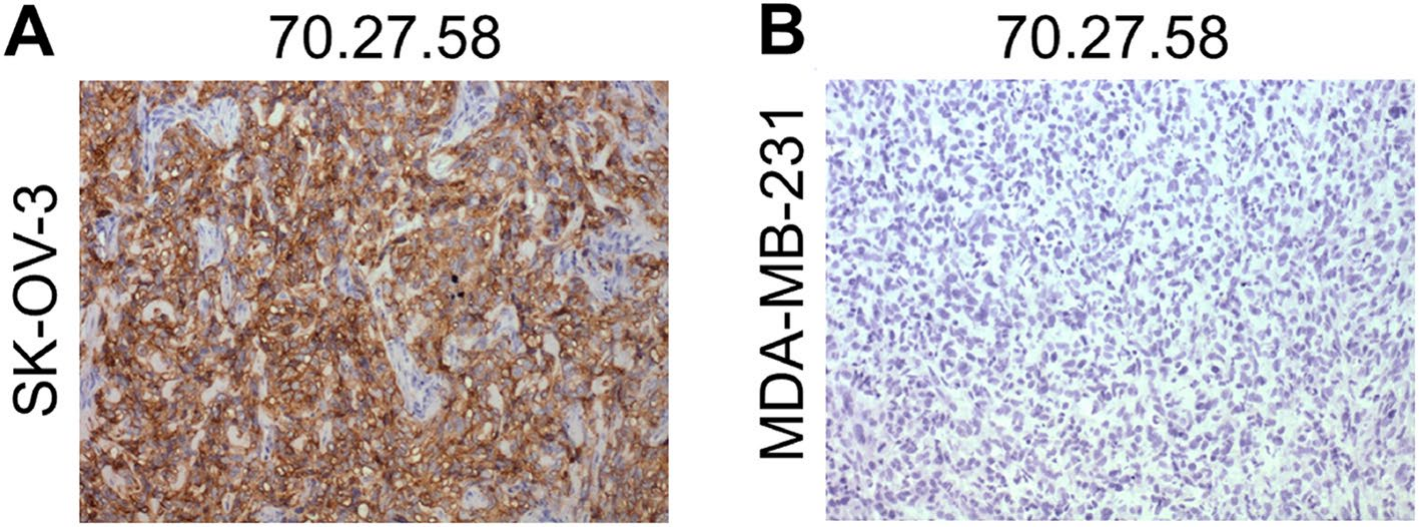
HER2 expression in tumors from mice with xenografted human cancer cells. (**A**) Immunohistochemistry staining using anti-HER2/70.27.58 mAb of the mouse tumors induced with the human ovarian cancer cells (SK-OV-3) overexpressing HER2 and (**B**) human epithelial breast cancer cells (MDA-MB-231) with low expression of HER.

Animal models are often used for studying molecular mechanisms of HER2-associated cancers and drug development. The appropriate assay for HER2 quantification in different tissues is desired. Therefore, we have tested the serum and tumor tissue samples obtained from mice with xenografted human cancer cells to verify the applicability of the newly developed assay for the determination of the human HER2 in the mouse model. For this purpose, tumors were generated by inoculation of animals with human ovarian cancer cells (SK-OV-3; HER2-positive, group A) or human epithelial breast cancer cells (MDA-MB-231; HER2-negative, group B). The HER2 receptor status of the mouse tumor was assessed by immunohistochemistry with anti-human HER2 antibody. As expected, the lesions formed in mice inoculated with SK-OV-3 cells revealed intense human HER2 expression (Fig. 4A; Fig. S4), in contrast to the mice receiving MDA-MB-231 cells that were scored as negative for the staining with anti-HER2 antibody (Fig. 4B).

The serum was also collected from the experimental mice of both groups A (SK-OV-3 tumors overexpressing HER2) and B (MDA-MB-231 tumor with low HER2 expression). The ELISA measurements showed a significant amount of HER2 (129.5–166.1 ng/ml) in group A (Table 3). In contrast, HER2 protein was undetectable in serum from mice of Group B.

**Table 3:** HER2 levels in the mice sera and tumors from mice xenografted with human cancer cells.

Sample	Group A (SK-OV-3)			Group B (MDA-MB-231)		
	Mouse 1	Mouse 2	Mouse 3	Mouse 1	Mouse 2	Mouse 3
**Serum** soluble HER2	162.1 (4.6%)	129.5 (6.6%)	166.1 (7.6%)	< LOD	< LOD	< LOD
**Tumor** soluble HER2	80 825.0 (4.4%)	50 778.1 (3.4%)	10 102.9 (5.7%)	< LOD	< LOD	< LOD
**Tumor** cell-bound HER2	101 625.4 (4.8%)	41 389.2 (8.6%)	82 186.4 (1.6%)	< LOD	< LOD	< LOD

The samples of blood and tumors from mice with xenografted SK-OV-3 (group A, HER2 overexpressing) or MDA-MB-231 cells (group B, HER2 low expression) were tested using the sandwich in-house ELISA. Average values of HER2 concentrations represent results from 2 ELISA measurements performed in triplicates and are expressed in ng/ml along with the coefficient of variation shown in brackets (%CV).

HER2 was also quantified in the corresponding tumor tissue from the same mice. The sample extracts containing PBS-soluble proteins and the whole tissue proteins soluble in RIPA buffer were tested by in-house ELISA. In comparison to the serum, the HER2 levels in the tumor tissues in group A were several times higher, however, it varied depending on the individual mouse (Table 3). The concentration of soluble HER2 (PBS-extracted) was in the range of 10.1–80.8 $\upmu$g/ml, while the cell-bound HER2 levels (RIPA lysates) were observed in the range of 41.4–101.6 $\upmu$g/ml. At the same time, the level of HER2 in all tested samples from Group B was below the limit of detection (< LOD).

Our results confirmed the usability of the developed ELISA for quantification of HER2 protein that is released into the blood serum or the extracellular matrix (PBS extract) as well as the protein bound to the cell membrane (RIPA lysate). The assay can also distinguish the cells that overexpress HER2 (in vitro and in vivo) from the cells that are considered HER2-negative. All measurements were of satisfactory quality with the %CV below 10%.

#### HER2 determination in human serum

The in-house developed ELISA was used on the human serum sampled from patients. The study included samples from 9 female patients with confirmed breast cancer and 1 patient with non-cancerous endometrial pathology. The HER2 control sample constituted the serum from a pooled blood of 3 donors with no reported pathology (PB-pool). The receptor status (including estrogen, progesterone, and HER2 receptors) of the tumors from corresponding cancer patients was determined by IHC (and FISH if necessary). The cohort included HER2 low and ultralow expression cancers were considered HER2 negative, i.e. HER2 (1+) or HER2 (2+/FISH negative) with either hormone receptor-positive (ER 3+, PR 3+) or negative (ER -, PR -) status (Table 4).

**Table 4:** HER2 measurements in the human serum samples from patients and control donors.

No	Receptor status of tumors corresponding to the tested serum	Average level of HER2 [ng/ml]		
		Sandwich ELISA—initial testing *	Sandwich ELISA—comparison testing ^@^	R&D Systems—comparison testing ^@^
1	Breast cancer ER-/PR-/HER2(1+)	181.4 (5.07%)	–	10.7 (0.1%)
2	Breast cancer ER-/PR-/HER2(1+)	71.9 (5.37%)	–	4.9 (2.6%)
3	Breast cancer ER(3+)/PR(3+)/HER2(1+)	183.7 (1.75%)	18.3 (0.42%)	11.7 (16.1%)
4	Breast cancer ER(3+)/PR(3+)/HER2(1+)	> 210.0 (3.7%)	–	9.9 (23%)
5	Breast cancer ER(3+)/PR(3+)/HER2(2+)/FISH negative	161.4 (0.02%)	–	10.6 (16.8%)
6	Breast cancer ER(32/PR(3+)/HER2(1+)	165.3 (4.78%)	–	13.7 (2.9%)
7	Breast cancer ER(3+)/PR(3+)/HER2(1+)	129.8 (11.63%)	–	8.2 (8.5%)
8	Breast cancer ER(3+)/PR(3+)/HER2(1+)	195.3 (7.33%)	18.7 (1.07%)	12.6 (3.6%)
9	Non-cancerous pathology of endometrium	83.8 (4.31%)	8.6 (1.31%)	10.1 (15.7%)
10	No pathology (PB-Pool)	29.5 (1.85%)	17 (1.83%)	21.8 (4%)

The average level of ECD HER2 in ng/ml was calculated from two independent ELISA measurements with 3 replicates for each sample; the coefficient of variation is shown in brackets (%CV). A result > 210.0 exceeds a test sensitivity resulting in compromised statistical analysis. The last two columns show a comparison of results acquired with the R&D Systems kit and newly developed ELISA.

*Initial analysis performed on serum stored for short-term at -20 °C.

^**@**^Analysis performed on serum samples stored for over a year at -20 °C.

Before analysis, the serum samples were diluted 2 times in the PBS, following the optimized ELISA protocol. Our results showed a variable amount of HER2 ECD in the serum with the concentration ranging from 71.9 to above 210 ng/ml in the patient with breast cancer showing low expression of HER2 (1+), which is considered HER2 negative tumor (Table 4). In the same range was the HER2 level (83.8 ng/ml) in the serum of the patient with a benign pathology of endometrium. In contrast, the HER2 ECD serum level in the control pooled sample was at a low concentration of 29.5 ng/ml. This is slightly above values which are considered for individuals with no evidence of cancer disease (below 22 ng/ml)^4^. To prove that the results obtained with the newly developed ELISA are reliable we have employed for sample testing the Quantikine ELISA Human ErbB2/Her2 kit, commercially available from R&D Systems (USA, Minneapolis). Comparison analysis was performed on 4 serum samples previously stored at −20 °C for about a year. Both tests, i.e. the commercially available and newly developed in-house ELISA showed consistent results when tests were conducted at the same time point (after long-term storage at −20 °C). The precision of the developed sandwich ELISA is also demonstrated by the %CV value obtained for the tested samples. Only for one serum sample it exceeded the 10% threshold and amounted to 11.63%. For comparison, the %CV for the commercial ELISA from R&D Systems was above 15% for 4 of 10 samples tested. A significant decrease in the HER2 level was noticed in samples tested initially and after long-term storage. All results are summarized in Table 4.

## Discussion

HER2 is overexpressed in several aggressive cancers, including gastric and breast cancer. After shedding the extracellular domain the receptor is released into the blood and the concentration of the soluble form of the protein fluctuates over time^16^. As the level of sHER2 ECD is associated with the clinicopathologic parameters and disease-free survival (DFS), its prognostic value in metastatic breast cancer has been suggested for a while now^16–20^. This biomarker is also considered a predictive factor in patients undergoing HER2 targeting immunotherapies^21^. Increasing awareness of the HER2 importance in carcinogenesis is followed by the development of HER2-targeting agents, diagnostic tools, and individualized therapies^22^. Fast and precise HER2 level measurement has vast potential for clinical use and with the emergence of developing diagnostic tools, this study presents yet another detection approach focusing on HER2 biomarker. While among HER2 examination methods FISH and IHC are the gold standard in clinical diagnostics, they rely on tissue biopsy, inherent with heterogeneity of the tumor mass and subjective image analysis (IHC) of specimens stained with probes detecting HER2. Thus, we developed novel mouse anti-HER2 monoclonal antibodies recognizing the epitope located on the ECD domain. It allows for the determination of HER2 biomarker not only located on the cell membrane, but also in the secreted form that is released from the tumor into the extracellular matrix, circulation, and in vitro—into the culture medium. The obtained clones of monoclonal antibodies were verified for specificity by several means and compared to the commercial antibody counterparts raised in different hosts (Fig. 2D, E, and S1).

The unique CDR domains of the heavy and light immunoglobulin chains of distinct antibody clones were confirmed by nucleotide sequence analysis (Fig. 1A) supporting the binding of different epitopes within the HER2 ECD. It provides an opportunity for the design and standardization of sandwich ELISA with a great possibility to be employed in a non-invasive serum-based assay measuring HER2 biomarker^23^. The unique feature of our assay comes from a specific tandem of monoclonal antibodies binding simultaneously to the same molecule of HER2 protein. The assay with its simplicity of performance and reduced time of the analysis generates results that allow for objective quantification of the HER2 protein released from cells and circulating in the blood. Furthermore, the native state of the detected antigen is not limiting when using these tools, as monoclonal antibodies bind also the denatured form of HER2 protein, shown on WB of the cancer cell lysates probed with the anti-human HER2/70.27.58 mAb (Fig. 2D) and IF of the formaldehyde-fixed cells. The affinity of the capturing antibody (immobilized on the plate) is sufficient to reach the detection level required for quantification of HER2 in a variety of samples, including cell cultures (Table 2), tumor tissue (Table 3), mouse and human blood (Table 3, 4). Thus, the developed ELISA can be easily used for research purposes on a variety of models, i.e. to study HER2's role in cancer and therapy. More study is still required to confirm the correlation of the serum HER2 with the disease stage or treatment outcome. The quantification of the HER2 biomarker using ELISA on the biopsied tumor samples can be more objective in contrast to the IHC staining analysis. We have shown that the assay can be used for HER2 determination in the tumor tissue of the human cells-xenografted mice (Table 3), however future studies on the adequate number of human tumor samples will be necessary to confirm its applicability for clinical diagnostics.

Assay validation is of special importance when considering utility in diagnostic purposes. Sandwich ELISA in general can be used for virtually all liquid-based biological sample types. During the process of ELISA development, a spike and recovery (SAR) assay performed on a variety of matrices (PBS, human serum, FBS, cell lysates, and culture medium), tested with mocked samples of known HER2 concentration revealed generally good recovery with only a slightly worse performance in FBS and some SK-OV-3 medium and whole cell lysates. These results fit into the accepted recovery range of 80–120%^15^. An unexpected rise in signal strength was observed for the sample of the pooled human serum (female only). For all tested HS samples the values are increased by 11–19 ng/well, which suggests the presence of some interfering factors. Sample dilution allowed for mitigating this negative effect. Heterophilic antibodies are poorly understood polyreactive human antibodies, that recognize IgG from different species and are often mentioned in literature as the cause of diagnostic failures in immunoassays^24^. These antibodies have no clear immunogen and are of more non-specific type^25^. In the sandwich-type immunoassays, they can form a bridge between the capture and detection antibodies, leading to a false-positive result in the absence of analyte or increased readout, in case of target presence. In contrast, false negative results due to the interference of heterophilic antibodies are rarely observed^26^. Taking into account the mechanism in which heterophile antibodies influence the increase in the obtained results, it is highly probable that they are present in the human serum used as the matrix for the SAR assay. Additional testing is needed to define whether this effect is also observed in the individual patient sera. However, the observed higher level of HER2 in the pooled serum, after long-term storage of samples in the measurements performed in parallel using the commercial and homemade assays was not prominent (Table 4). Nevertheless, diluting the tested sample for analysis provides an accepted solution to overcome the interfering factors.

Considering assay sensitivity, it is comparable with other commercial ones, like the FDA-approved and provided by Siemens Healthineers Diagnostics (LOQ of 0.5 ng/mL, a linear measuring range up to 350 ng/mL)^3^. We showed that our approach allows for quantification of HER2 biomarker in samples from human tumors developed in patients (Table 4) and xenografted mice (Table 3). The results of HER2 quantification in human serum performed in parallel with the available commercial assay, i.e. R&D Systems, showed similar HER2 levels and confirmed reliable measurement of the soluble HER2 in the blood (Table 4). Noticeably, the determined levels of HER2 protein changed over time with sample storage at −20 °C. It is in contrast with other reports concluding that ex vivo HER2 antigen remains stable when samples are stored refrigerated or frozen for several years^13^. However, we have analyzed the HER2 level by repeating quantification in the same samples (not comparing different sample sets stored for several years or used as fresh) and showed that the HER2 level significantly changed in the tested samples after one year at −20 °C. We assume that the signal decrease might be also a result of the different targeted epitope on the HER2 protein that in the case of our antibody might be less stable (especially after several cycles of freezing–thawing) than the epitope targeted by other HER2 antibody types. Since we have not performed antigen mapping for the developed antibody the issue with antigen stability warrants further investigation.

Importantly, our assay has been verified for HER2 biomarker quantification on the serum samples from breast cancer patients with the receptor status labeled as low (HER2 (1+) and HER2 (2+)/FISH negative). This type of diagnosis in the current ASCO recommendations on breast cancer diagnostic procedures falls into the HER2 negative cases^27^, for which anti-HER2 therapy was not recommended until recently. Last year the low-expressing HER2 cancers were reconsidered for the anti-HER2 therapy showing promise for the remaining half of all breast cancer patients with low HER2 expression^28^. However, the discordance still exists^29,30^ and new reliable diagnostic approaches are desired. The presented assay employing mAbs with the confirmed unique sequences of the CDRs show a potential for future clinical application in the assessment of HER2 status, based on the biomarker level in blood. As the comprehensive validation of the assay on clinical samples was not within the subject of this report, the diversity of tested samples shows limitations (Table 4 presents the tumor receptor status of patients included in this study). The assay performance evaluation in future studies will require employing a more representative and comprehensive cohort of patients, including HER2 3 + , triple-negative cases, and especially HER2-low tumors, as recently emerging new subtype of breast cancer.

Finally, for convenience, some assay technical aspects, like plate pre-coating and fixing for long-term storage, were incorporated into the process of assay procedure development. Preparation of the initial steps of the assay ahead of time brings several benefits for the ELISA performance. Firstly, using a pre-coated plate reduces the time of the experimental manipulation to ~ 5 h, thus the method can be considered as rapid with sample preparation to data processing and result interpretation, all within one day. Secondly, the preparation of several plates at the same time and storing them until use significantly limits the differentiation between samples analyzed at different times or between batches. Finally, performing critical coating steps all at once helps to unify analyses conducted in spatially different places, such as different laboratories. All these aspects find reflection in more precise, reproducible, and specific results that add to standardization of the clinical methods.

In summary, the unique combination of specific monoclonal antibodies and immunochemical assay presented in this study allows for a comprehensive determination of the cell-bound and soluble HER2 in different tissue samples of humans and mice. With a high specificity, accuracy, and reproducibility of detection of both endogenous and recombinant HER2 ECD, it provides insight into the future perspectives of target-specific diagnostic and predicting tools.

## Methods

### Hybridoma cell lines generation

Monoclonal antibodies specific to HER2 were obtained employing hybridoma technology^14^. The animal studies were performed according to the Interdisciplinary Principles and Guidelines for the Use of Animals in Research, Marketing, and Education issued by the New York Academy of Sciences Ad Hoc Committee on Animal Research and were approved by the Local Ethics Committee for Animal Experiments at Hirszfeld Institute of Immunology and Experimental Therapy, Wrocław, Poland (Permission No. 89/2018/P1). The study is reported following ARRIVE guidelines. A group of 5 Balb/c mice (Center for Experimental Medicine, Białystok, Poland) 5–8 weeks old, were immunized with the full-length CD340 protein as described earlier^31^, three times at two weeks intervals. Injections were formulated with aluminum oxide adjuvant, and the final injection (two days before euthanasia) was given intraperitoneally without adjuvant. Immunization progress was followed by the analysis of serum samples using ELISA and the best responding animals were chosen for spleen harvesting after euthanasia by carbon dioxide inhalation. Spleen cells were isolated and fused with myeloma cells Sp2/O-Ag14 using PEG, according to standard fusion protocol. The cloning was performed using a limiting dilution protocol. Clones were screened in an ELISA assay using plates coated with a recombinant HER2 protein fragment spanning the N-terminal amino acids from Thr23–Ala510 that constitute the extracellular domain of a native HER2 receptor, and purified from the HEK293 expression system (ProSci, Fort Collins, CO, USA). Additional tests were conducted on plates coated with recombinant HER2 His-tag (ProSci, Fort Collins, CO, USA) to ensure antibody specificity.

After the establishment of several monoclonal, stable lines, cultures were expanded to produce and purify mAbs. Finally, the two best-performing clones secreting anti-HER2 antibodies towards different epitopes of HER2 antigen, namely clones 70.27.58 and 70.21.73.67 were utilized in this study.

The unique nature of the selected anti-HER2 antibody clones has been confirmed by sequencing and nucleotide analysis within the light and heavy chain complementarity-determining regions (CDRs) in comparison to the known mouse anti-HER2 antibodies.

### Culturing of hybridoma cell lines for antibody production

Hybridoma cell lines were grown at 37 °C in the atmosphere of 5% CO_2_. The cells with mixed adherent and suspension character were initially kept in DMEM with high glucose and supplemented with 10% (v/v) FBS until reaching full confluence. Hybridoma cells in these conditions show approx. 70–80% viability. For the production step, the hybridomas were transferred onto a serum-free medium, Panserin H4000 (PAN-Biotech, Aidenbach, Germany). The best antibody yield was obtained under a dense culture condition, thus EZ flask (KDBio, Berstett, France) production was a preferred method. Upon 30 days of culture, the medium was collected, filtered, and stored at 4 °C until purification.

### Culturing of human cancer cells

Human cell lines of the breast cancer (MDA-MB-231, SK-BR-3) and ovarian cancer (SK-OV-3) used in this study came from the American Type Culture Collection (ATCC). Cells were grown at 37 °C, in a 5% CO_2_ atmosphere, as described earlier^32^. Briefly, the complete culture medium was based on DMEM with high glucose supplemented with 2 mM ʟ-glutamine, 10% (v/v) heat-inactivated FBS. Cells were grown on Petri dishes and passaged at ~ 80% confluence to a fresh medium. For passaging, cells were collected by trypsinization and transferred into separate 15 ml conical tubes and counted using a Burker chamber. The experimental cells were seeded into 12-well plates (3 × 10^5^ cells/per well) and allowed to attach overnight. Medium and cells for lysates were used in further analysis.

### Purification of the anti-human HER2 antibodies

For the anti-HER2 antibody purification, the NGC Medium-Pressure Chromatography System (Bio-Rad) was employed in the affinity chromatography. First, the HiTrap® Protein A High Performance (Cytiva, Marlborough, MA, USA) column was equilibrated with the Binding Buffer (0.2 M boric acid, 0.02 M sodium phosphate, 0.02 M sodium citrate dihydrate, 1.0 M sodium sulfate, pH 9.0). Next, the hybridoma medium was loaded onto the column, and upon a column wash with the Binding Buffer, the antibody was eluted from the column in a gradient of the Elution Buffer (0.02 M sodium citrate dihydrate, 0.1 M sodium chloride, pH 2.5). 1.5 ml fractions were collected throughout the elution. Anti-HER2 antibody was eluted from the column with ~ 60% of the Elution Buffer and was immediately neutralized with 75 µl of 3 M Tris–HCl pH 8.8. The antibody presence in the collected fractions was confirmed by protein concentration measurement using Nanodrop™ One instrument (ThermoFisher, Waltham, MA, USA), and the protein bands pattern was analyzed by SDS-PAGE. Fractions containing the highest concentration of the antibody were pooled and subjected to the buffer exchange to PBS, together with sample concentration using Amicon® Ultra Centrifugal Filter Units (Millipore, Burlington, MA, USA) with 50 kDa cut-off. The final quality and purity of the antibody sample were further tested by SDS-PAGE to confirm consistent preparations of batches and purity.

### Antibody sequencing

Cells were cultured in DMEM supplemented with 10% FBS to reach approximately 1 × 10^6^ cells. At the time of harvesting, cells were detached from the surface by mechanical scrapping, transferred to a sterile 1.5 ml centrifugal tube, and centrifuged for 5 min at 1 000 × g. The pellet was resuspended in 1 ml of TRIzol reagent by pipetting, and the samples were stored at −20 °C for shipping. The sequencing of the complementarity-determining regions (CDRs) was performed by ProMab Biotechnologies, Inc., Richmond, CA, USA to determine the unique sequence of the constant and variable regions of the generated antibodies.

### Immunohistochemistry

Immunohistochemistry (ICH) was performed as described earlier^33^. The tissue was fixed in 4% (v/v) formaldehyde in PBS at the time of surgical removal and was embedded in paraffin (FFPE) for cutting into 4 μm sections. The slices mounted on poly-*L*-lysine-coated glass slides were first deparaffinized by heating at 60 °C and then immersed in xylene for 9 min, followed by antigen retrieval in DAKO Retrieval Solution pH 9.0 by heating in microwave Daewoo at 350 Watt and cooling at room temperature. The immunostaining was performed using the Vectastain Abc kit (DAKO, Glostrup, Denmark) according to the Producer protocol. First, the endogenous peroxidase was blocked with the Peroxidase-Blocking Solution (DAKO, Glostrup, Denmark) for 10 min at room temperature, followed by washing 2 × with distilled water. After 15 min of blocking with Protein Block Serum-Free (DAKO, Glostrup, Denmark) at room temperature and washing with distilled water, the sections were incubated overnight at 4 °C with anti-HER2 antibody (Hercept Test, DAKO, Glostrup, Denmark). The sections washed with PBS were next subjected to 30 min incubation with EnVision + System-HRP (DAKO, Glostrup, Denmark) at room temperature and washed (2 × 5 min) with PBS. The reaction was developed with 3,3’-diaminobenzidine tetrahydrochloride (DAB, DAKO, Glostrup, Denmark) for 5 min. Finally, the slides were counterstained with hematoxylin–eosin (H&E) and mounted under coverslips. Pictures were taken using the Olympus BX51 microscope (Tokyo, Japan).

### Immunofluorescent staining

Cells were cultured on chamber slides and processed for immunofluorescent staining as described earlier^33^. Briefly, upon 3 washes with PBS, cells were permeabilized with 0.5% Triton X-100 for 15 min at room temperature, and the blocking step was performed using a blocking solution containing 3% NFDM, 1% BSA, 0.1% Triton X-100 in PBS for 30 min at room temperature. Chamber slides were next incubated overnight at 4 °C with a mixture of the rabbit mAb anti-HER2 (Abcam, Cambridge, UK)(0.5 µg/ml in the blocking solution) and either mouse mAb anti-HER2 clone 70.27.58 (0.5 µg/ml in blocking solution) or clone 70.21.73.67 (0.5 µg/ml in blocking solution). After 3 washes with PBS, the chamber slides were incubated for 1 h at room temperature with secondary anti-mouse IgG-AF488 (Thermo Fisher Scientific, Waltham, MA, USA) and anti-rabbit IgG-AF594 (Abcam, Cambridge, UK). Unbound antibodies were removed by 3 washes with PBS, followed by mounting the chamber slides with the mounting medium containing DAPI for nuclear staining. Control slides with a single antibody, as well as secondary antibody controls, were performed in parallel to exclude possible unspecific reactions. The slides were observed under the Nikon Eclipse Ti-2U Fluorescent microscope (Tokyo, Japan).

### Human serum samples

For the human serum testing, blood from clinical patients with confirmed breast cancer was collected at the time of the surgical removal of the lesion. The breast cancer patients were diagnosed by the core-needle biopsy and subsequent IHC staining for HER2 overexpression and the status of estrogen and progesterone receptors. In the case of ambiguous status of HER2 (2+) the FISH analysis was run. The tissue from the patient with non-malignant endometrium pathology was not tested by IHC for receptor status since it is not a routine practice in case of non-cancerous tissue changes. The collected blood was processed for serum and stored at − 20 °C until analysis. The study was conducted according to the guidelines of the Declaration of Helsinki and approved by the Bioethical Committee of the Medical University of Lublin (KE-0254/50/2015). Informed consent was obtained from all subjects. Blood from the control patient (non-malignant endometrium pathology) was used as the control in addition to a commercially available pooled serum derived from the blood of 3 female donors aged 39, 60, and 70 years with no reported pathology (NeoBiotech, PB-Pool-150 mL-20211214) as a reference.

### Xenograft mouse model

All experiments were performed according to the Interdisciplinary Principles and Guidelines for the Use of Animals in Research, Marketing, and Education issued by the New York Academy of Sciences Ad Hoc Committee on Animal Research and were approved by the 1st Local Committee for Experiments with the Use of Laboratory Animals, Wrocław, Poland (Permission No. 05/2017) and are reported following ARRIVE guidelines. Xenograft mouse model of the human HER2 positive and HER2 negative tumors, each group of 3 mice, was developed on 6–7-week-old CB-17-PrkdcScid (SCID) female mice provided by Janvier Labs, France, and maintained in specific pathogen-free conditions. Mice were inoculated subcutaneously in the right body side with tumor cells SK-OV-3 (group A) or MDA-MB-231 (group B) (s.c; 10 × 10^6^ of SK-OV-2 cells and 6 × 10^6^ of MDA-MB-231 cells /0.2 ml matrigel + PBS, 2:1; BD Matrigel Basement Membrane Matrix High Concentration, Corning). When tumors became palpable, their maximum length and width were measured by caliper, and the tumor volume was calculated using the formula: (a^2^ x b)/2, where a = short axis length in mm and b = long axis length in mm. The tumor was allowed to reach 81.1–669.5 mm^3^ before excision. The animals were euthanized with Buprenorphine (0.1 mg/kg, s.c.) and decapitation before collection of the tissue samples.

Serum samples were obtained from centrifugation of full blood drawn after removal of the tumor and stored at −20 °C until testing. Cell lysates from the dissected tumor tissue were obtained by maceration, of the tissue sample crushed mechanically with a glass rod in the dry ice ethanol bath. Tumor samples were rinsed twice with PBS (tumor–soluble HER2), and afterward, three times with PBST followed by final cell lysis performed with the RIPA Buffer (Sigma-Aldrich, Saint Louis, MO, USA) and mechanical homogenization by running the lysate 5 times through the 21G injection needle (dispoFINE), aiming to extract membrane-bound proteins (tumor—cell-bound HER2). Protein Inhibitor Cocktail (Sigma Aldrich, Saint Louis, MO, USA) was added to all used buffers. Collected samples were centrifuged for 10 min at 14 000 × g at 4 °C, and the supernatant was transferred to a new tube, for storage at −20 °C until testing. In addition, to confirm HER2 expression, half of the excised tumor tissue was subjected to immunohistochemistry as described earlier for human tumors^33^.

### Direct ELISA

A direct ELISA was used for the evaluation of the developed monoclonal antibodies specific to HER2 ECD. A flat bottom 96-well plate (MaxiSorp, Nunc, Thermo Fisher Scientific, Waltham, MA, USA) was coated with the Human HER2 Protein Fc Tag chimeric protein (AcroBIOSYSTEMS, Newark, DE, USA) diluted in 0.1 M NaHCO_3_, pH 9.6 buffer at 0.1 µg/well and incubated overnight at 4 °C. After 4 washes with PBST using an Immunowash Microplate Washer (Bio-Rad, Hercules, CA, USA), the plate was blocked from non-specific binding with 5% (w/v) NFDM in PBS. Incubation was performed for 1 h at 37 °C and was followed by 4 washes with PBST. Next, a series of monoclonal antibody anti-human HER-2/70.27.58 or anti-human HER2/70.21.73.67 dilutions were allowed to bind the antigen for 1 h incubation at 37 °C, followed by 4 times washing with PBST. After 1 h incubation at 37 °C with secondary goat anti-mouse antibody conjugated with HRP (Abcam, diluted 1:20 000 in PBS) and 4 washes with PBST, the plate was incubated with 3,3′,5,5′-tetramethylbenzidine (TMB; Abcam, Cambridge, UK) substrate, followed by addition of the STOP solution (Abcam, Cambridge, UK). The colorimetric signal was read at 450 nm using ELISA Reader BioTek 800 TS (BioTek Instruments; Agilent, Winooski, VT, USA). Collected data were analyzed using Gen5 IVD software (BioTek Instruments; Agilent, Winooski, VT, USA) and were expressed upon background subtraction as A_450_-A_0_.

### Sandwich ELISA development and validation

At first, the plate coating with the in-house developed anti-human HER2/70.27.58 mAb (SDS Optic Inc., Lublin, Poland), serving as capturing antibody, was tested in three tenfold concentrations: 0.01, 0.1, and 1.0 µg/well. In the final setup, the plate was coated with the capture antibody at 0.1 µg/well in NaHCO_3_, pH 9.6 buffer, by overnight incubation at 4 °C. After 4 washings with PBST, the surface blocking with the unrelated proteins with NFDM solution in PBST for 1 h at 37 °C was performed. In the optimization process two commonly used blocking agents: 2% Bovine Serum Albumin (BSA) in Phosphate-buffered Saline (PBS) and 5% NFDM in PBS with 0.05% Tween-20 (PBST), were tested. In the assay stability test the plate was incubated with 100 µl of 5% (w/v) trehalose in the blocking solution, for 1 h at room temperature. After the incubation, and the solution removal the plate was dried out from the remaining solution at room temperature and stored at 4 °C in humidity-controlled conditions for 4 months, to assess the loss of the assay activity.

To test the samples of interest containing a target analyte it was applied on the plate (100 $\upmu$l/ well, diluted in PBS) and incubated for 1 h at 37 °C. After the washing step with PBST, the biotin-labeled detection antibody (anti-human HER2/70.21.73.67 mAb, SDS Optic Inc, Lublin, Poland) was added and incubated for 1 h at 37 °C. The optimization of the biotinylation procedure of the detection antibody was performed using two different commercially available kits (Abnova, Taipei, Taiwan, and Invitrogen, ThermoFisher Scientific, Waltham, MA, USA). The reactivity of the labeled detection antibody was compared for avidin-HRP conjugates (ThermoFisher Scientific, Waltham, MA, USA; at dilution 1:40,000 and 1:80,000 in PBS) and streptavidin-HRP (Merck Millipore, Burlington, MA, USA; at 1:2000). Next, the selected avidin-conjugated horseradish peroxidase was applied and incubated for 1 h at 37 °C. The plate was washed 4 times with PBST in between incubations. TMB (3,3′,5,5′-tetramethylbenzidine; Abcam, Cambridge, UK) was used as the substrate followed by STOP solution (Abcam, Cambridge, UK) to terminate the reaction between peroxidase and TMB substrate. For results calculation, the absorbance was read in ELISA Reader BioTek 800 TS (BioTek Instruments, Inc., Agilent, Winooski, VT, USA) at 450 nm (absorbance filter) and expressed after background subtraction. Collected data were analyzed using Gen5 IVD software (BioTek Instruments, Inc., Agilent, Winooski, VT, USA). The concentration of HER2 protein in the analyzed sample was calculated based on the standard curve established with the recombinant HER2 protein Fc Tag (Acro BIOSYSTEMS, Newark, DE, USA) loaded on the plate at the concentration of 1.56–100 ng/ml.

Assay sensitivity was determined on seven levels of spiked solutions ranging from 0.195 ng/ml to 1 ng/ml each in 6 replicates and two matrices, namely PBS and commercial pooled human serum. The lower limit of detection (LLOD) was defined as the concentration of HER2 that could be distinguished from the concentration of the blank control. Testing for the lower limit of quantification and upper limit of quantification (LLOQ and ULOQ) was conducted on matrices as for LLOD. Seven levels of spiked solutions ranging from 0.78 ng/ml to 300 ng/ml, each in 3 replicates were evaluated.

Assay linearity was assessed on mocked samples for low (2, 5, 10 ng/ml), medium–high (30 ng/ml), and significantly increased (50 ng/ml) concentrations of the antigen in PBS. The coefficient of intra- and inter-assay variations were studied on different days under the same operating conditions on replicates of at least 12 assays.

### Spike and recovery assay (SAR)

SAR was used as a variant of a standard ELISA procedure. A known amount of analyte (recombinant HER2 ECD Fc Tag protein) was spiked into the tested matrix (PBS, HS, FBS, or MDA-MB-231 and SK-OV-3 medium/whole cell lysate) after the blocking step. Concentrations of 2, 5, 10, 30, and 50 ng/ml of recombinant HER2 were prepared for each matrix. After 4 washings with PBST, the reaction was followed with biotinylated antibodies. The assay was run to measure the response (recovery) of the spiked sample matrices compared to HER2 in the standard curve diluent (PBS).

### Protein concentration determination

Protein concentration was determined using the Bradford assay protocol (Bio-Rad Laboratories, Hercules, CA, USA), based on a standard curve generated with BSA.

### Statistical analysis

All statistical analyses were performed using R 4.1.2 for Windows Software. R is an open-source project that is distributed under the GNU General Public License^34^. The mean value and coefficient of variation (%CV) of the OD_450 nm_ after background subtraction (A_450_-A_0_) were calculated by statistical analysis. The Shapiro–Wilk test for normality, Spearman’s rank correlation test, Pearson’s product-moment correlation test, F test to compare two variances, and two-sample t-test were used for data analysis. P-value < 0.05 was considered statistically significant in all the experiments.

### Supplementary Information

Supplementary Information.

## Data Availability

Nucleotide and amino acid sequences of heavy and light chains of the anti-human HER2/70.27.58 (GenBank: OR400146—https://www.ncbi.nlm.nih.gov/nuccore/OR400146, OR400147—https://www.ncbi.nlm.nih.gov/nuccore/OR400147) and anti-human HER2/70.21.73.67 (GenBank: OR400148—https://www.ncbi.nlm.nih.gov/nuccore/OR400148 and OR400149—https://www.ncbi.nlm.nih.gov/nuccore/OR400149) mouse monoclonal antibodies are available in the GenBank database.
