# Supplementary material for: The unique monoclonal antibodies and immunochemical assay for comprehensive determination of the cell-bound and soluble HER2 in different biological samples
Source: Sci Rep. 2024 Feb 17;14:3978. doi: 10.1038/s41598-024-54590-z (PMC10874376; doi:10.1038/s41598-024-54590-z)
Supplement: Supplementary file 1 — Supplementary Information. [file 41598_2024_54590_MOESM1_ESM.docx]

**The unique monoclonal antibodies and immunochemical assay for comprehensive determination of the cell-bound and soluble HER2 in different biological samples**

Aleksandra Antos^1,&^, Agnieszka Topolska-Woś^1,&^, Marcin Woś^1^, Agata Mitura^1^, Paulina Sarzyńska^1^, Tomasz Lipiński^2^, Andrzej Kurylcio^3^, Piotr Ziółkowski^4^, Marta Świtalska^5^, Joanna Tkaczuk-Włach^6^, Andrzej Gamian^7^, Wojciech P. Polkowski^3^, Magdalena Staniszewska^1,8*^

^1^ SDS Optic, EcoTech Complex, Block A, Głęboka 39, Lublin, 20-612, Poland

^2^ Lukasiewicz Research Network – PORT Polish Center for Technology Development, Stabłowicka 147, 54-066 Wrocław, Poland

^3^ Department of Surgical Oncology, Medical University of Lublin, Radziwiłłowska 13, 20-080 Lublin, Poland

^4^ Department of Pathomorphology, Wrocław Medical University, Marcinkowskiego 1, 50-368 Wroclaw, Poland

^5^ Department of Experimental Oncology, Hirszfeld Institute of Immunology and Experimental Therapy, Polish Academy of Sciences, Weigla 12, 53-114 Wrocław, Poland

^6^ Chair of Obstetrics and Gynecology, Faculty of Health Sciences, Medical University of Lublin, Staszica 4/6, 20-081 Lublin, Poland

^7^ Laboratory of Medical Microbiology, Hirszfeld Institute of Immunology and Experimental Therapy, Polish Academy of Sciences, Weigla 12, 53-114 Wrocław, Poland

^8^ Faculty of Medicine, The John Paul II Catholic University of Lublin, Konstantynów 1J, 20-708, Lublin, Poland

^&^ Authors contributing equally

*Correspondence: mstaniszewska@sdsoptic.pl

**Methods**

*Sandwich ELISA protocol for HER2 determination*

The clone 70.27.58 is immobilized on a plate as a target capturing antibody (1 µg/ml in 0.1 M NaHCO_3_, pH 9.6 buffer, 100 µl/well) by overnight incubation at 4°C (Fig. S2, Step 1). Antibody immobilization is than followed by 1 h incubation at room temperature on a horizontal microplate shaker. Total of four washes with PBS with 0.05 % Tween-20 (PBST, 400 µl each) is carried out between every incubation step. After surface blocking with the unrelated proteins (5 % non-fat dry milk solution in PBST, 100 µl/well) the HER2 analyte in the tested sample, including human serum (2 times diluted in PBS) and cell culture medium (samples with high HER2 concentrations require dilution in PBS), in the volume of 100 µl/well is loaded and allowed for binding for 1 h at 37°C (Fig. S2, Step 2). Along with the tested samples, a reference protein (recombinant chimeric protein composed of the human HER2 ECD fused to the human Fc fragment) is loaded in the serial dilutions within a range of 1.56 – 100 ng/ml (100µl/well, in triplicate) to facilitate standard curve generation. In the following step the second complementary anti-HER2 antibody (detecting antibody), binding a distinct epitope on the HER2 ECD fragment (clone 70.21.73.67) is applied (0.001 µg/µl in PBS, 100 µl/well), and incubated on a plate for 1 h at 37°C (Fig. S2, Step 3). Prior to analysis, this antibody is labeled with biotin to serve as a detector. Next, the applied biotin-labeled antibody forms a complex with an added avidin-HRP (1:40000 dilution in PBS, 100 µl/well, 1 h at 37°C incubation) and allowed for signal amplification (Fig. S2, Step 4). Substrate solution (100 µl/well) is added followed by 1 – 10 minutes incubation at 37°C, until the color changes to the expected level. A classical colorimetric substrate-and-enzyme reaction generates a signal that is proportional to the amount of HER2 protein bound from the tested sample (Fig. S2, Step 5). Stop solution (100 µl/well) is than added to terminate the reaction and optical density is determined using a microplate reader at the wavelength of 450 nm.

**Supplementary figures**

***
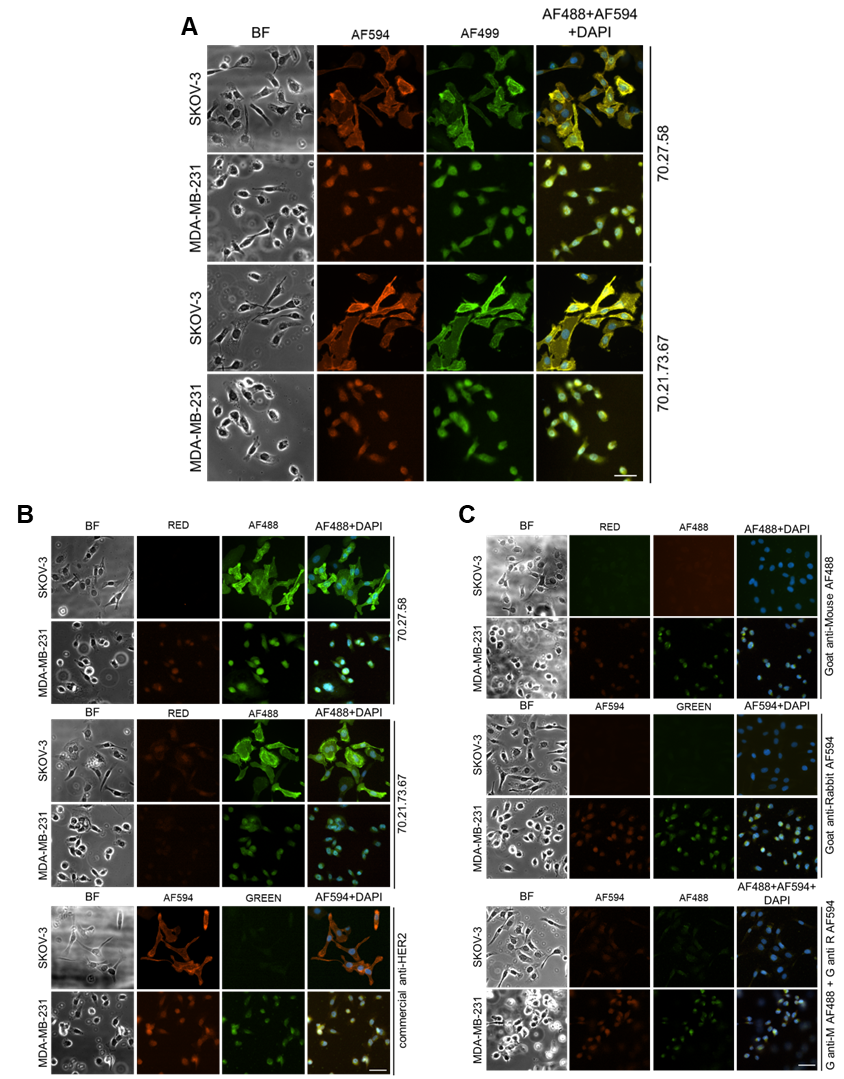
***

**Figure S1. Immunofluorescent staining of MDA-MB-231 and SK-OV-3 cells with anti-HER2 antibodies.** The co-staining was performed with **(A)** mixture of the commercial rabbit anti-HER2 mAb with either mouse mAb anti-HER2 clone 70.27.58 or 70.21.73.67), followed by anti-rabbit IgG conjugated with AlexaFluor594 (AF594, red) and anti-mouse IgG conjugated with AlexaFluor488 (AF488, green). Staining of cells with individual mouse (clone 70.27.58 or 70.21.73.67) and rabbit (commercial) antibody anti-HER2 followed by the appropriate secondary antibody used in panel A is shown in panel **(B)**; signal from AlexaFluor594 (AF594) and AlexaFluor488 (AF488) as red and green channel, respectively is shown as a background control. **(C)** negative controls of appropriate secondary antibodies: anti-mouse IgG conjugated with AlexaFluor488 (AF488, green), anti-rabbit IgG conjugated with AlexaFluor594 (AF594, red) and a mixture of thereof. VectaShield with DAPI was used to visualize nuclei of the cells. All slides were photographed in bright field (BF) or appropriate fluorescence filter using Nikon Eclipse Ti2-U Fluorescent microscope. Scale bar represents 50 µm.

**
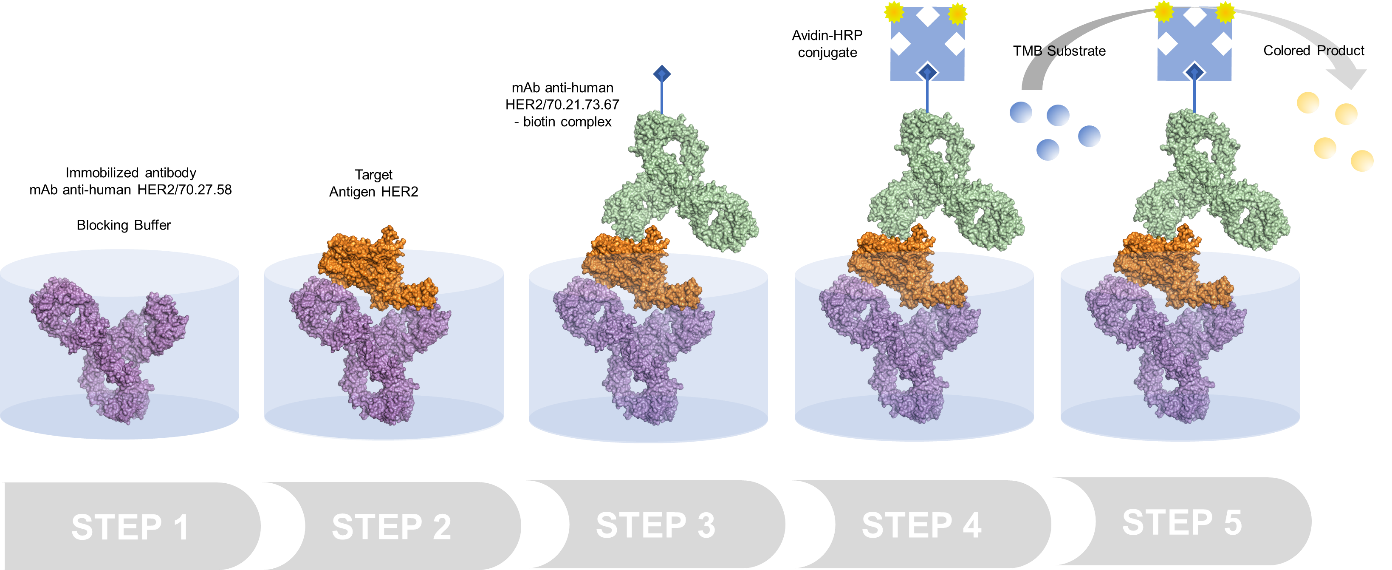
**

**Figure S2. Schematic diagram of the developed anti-HER2 sandwich ELISA protocol. Step 1** – immobilization of capturing anti-HER2 antibody; **Step 2** – interaction of the target analyte with the capturing antibody; **Step 3** - binding of the detecting anti-HER2 antibody labeled with biotin; **Step 4** – binding avidin-HRP for signal amplification; **Step 5** – development of the colorimetric substrate-and-enzyme reaction. Model structure of an IgG based on pdb entry: 5dk3 and HER2 ECD: 1n8z generated using PyMOL.

**
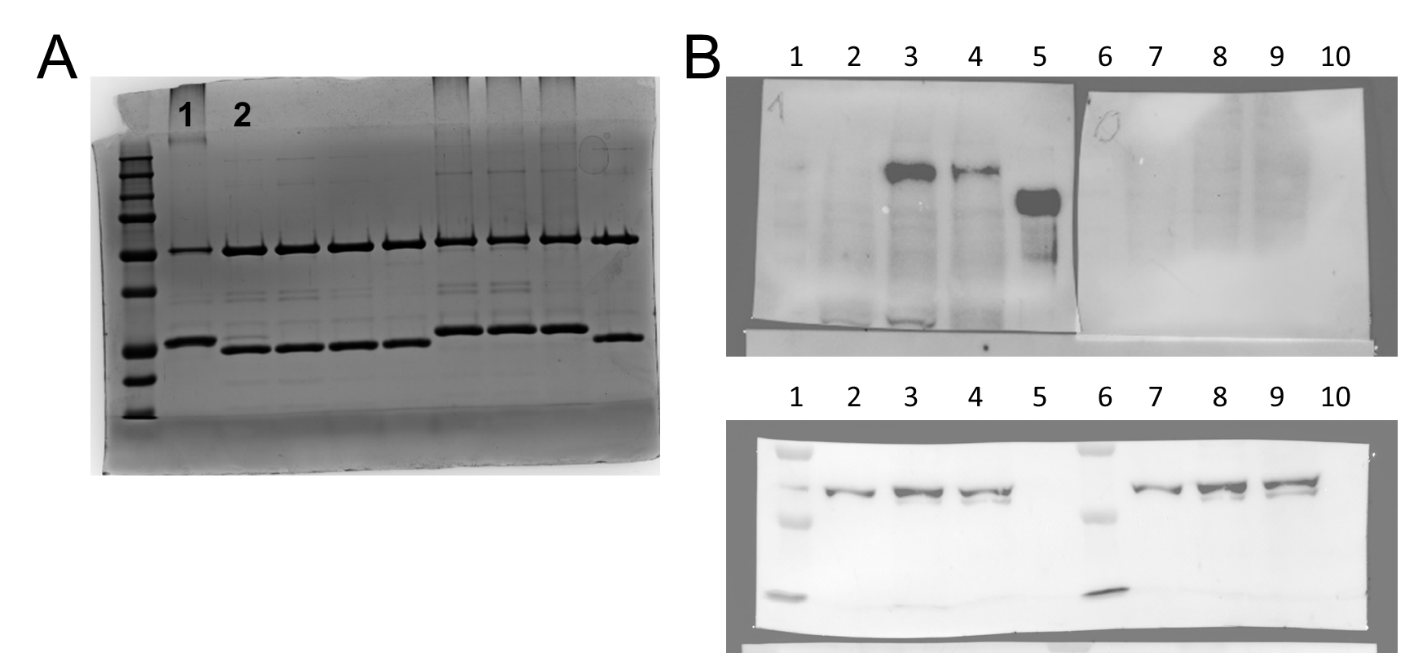
**

**Figure S3. Original SDS-PAGE (A) and WB (B) pictures used in Fig. 2C and D. (A)** Lines 1. and 2. correspond to the mAb anti-human HER2 clones 70.27.58 and 70.21.73.67, respectively. 12% SDS-PAGE without contrast control. **(B)** Lines 1 – 5 correspond to the molecular marker mass standards and whole cell lysate from MDA-MB-231, SK-BR-3, SK-OV-3, and recombinant HER2 ECD. Lines 6 – 10 represent the same amounts of molecular marker mass standards and the whole cell lysate as in lines 1 – 5 but where incubated with secondary antibody only, as a negative control of WB. The bottom panel represents the results for β-actin levels in respective samples.

**
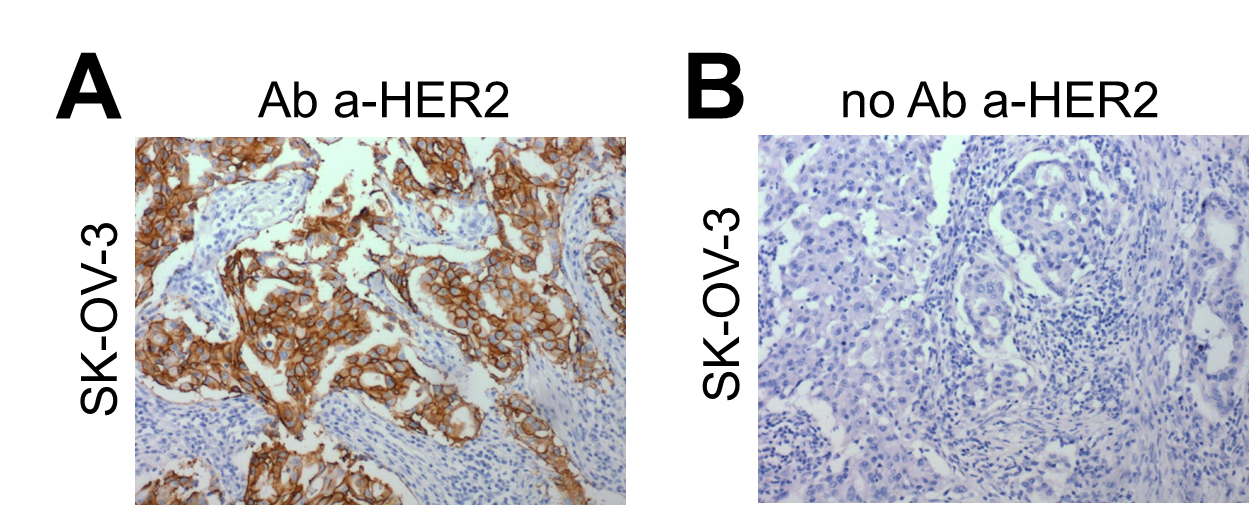
**

**Figure S4. Negative control for IHC staining shown in Fig. 5A.** The same tissue from the HER2+ tumor generated from xenografted SK-OV-3 cells was stained with **(A)** anti-HER2 antibody clone 20.27.58 or as a negative control **(B)** without anti-HER2 antibody.
